# Relationships between activity and well‐being in people with parkinson's disease

**DOI:** 10.1002/brb3.976

**Published:** 2018-04-16

**Authors:** Felicitas Ehlen, Katharina Schindlbeck, Lisa Nobis, André Maier, Fabian Klostermann

**Affiliations:** ^1^ Department of Neurology Charité ‐ University Medicine Berlin, CBF Berlin Germany; ^2^ Faculty of Psychology and Neuroscience Maastricht University Maastricht Netherlands

**Keywords:** chronic disease, nonmotor symptoms, Parkinson's disease, physical activity

## Abstract

**Objectives:**

The complex symptomatology of Parkinson’ disease (PD) usually goes along with reduced physical activity. Previous studies have indicated positive effects of activating therapies on patients’ well‐being. This study, therefore, examined how activity in daily life is related to patients’ subjective condition.

**Materials and Methods:**

Twenty‐one PD patients rated their condition every two hours during two routine days and documented the duration and type of their activities (based on the PRISCUS‐Physical Activity Questionnaire) during the respective time intervals. They were furthermore assessed regarding motor and nonmotor symptoms, personality factors, and coping strategies.

**Results:**

Patients spent on average 8.59 ± 2.93 hr per day at physical rest and 5.47 ± 2.93 hr physically active. We found highly significant associations between positive condition ratings (such as happiness, motivation, and concentration) and the duration of subsequent physical activities (*adj.r* ^*2*^ = .689) as well as between the duration of these activities and a subsequent improvement in the subjective condition (*adj.r* ^*2*^ = .545). This was strongest in patients using active coping strategies and showing agreeable and conscientious personality traits (*adj.r* ^*2*^ = .380). Nonmotor symptom severity was weakly inversely related to the daily amount of activities (*adj.r* ^*2*^ = .273), whereas no significant association with motor symptom severity was found.

**Conclusions:**

The results suggest a feedback process between a positive subjective condition and physical activities in PD patients. This appears to depend on the use of active coping strategies and nonmotor symptoms rather than on motor symptom severity. The results should encourage physicians to address the importance of everyday physical activities and to provide patients with behavioral advice.

## 1 INTRODUCTION

Parkinson's disease (PD) is one of the most frequent neurodegenerative movement disorders with an age‐specific increase and widely ranging estimates on prevalence (about 100 to 1000 per 100,000) and incidence (about 10 to 230 per 100,000 persons per year) in western European countries (von Campenhausen et al., 2005; Nerius, Fink, & Doblhammer, 2017). Stemming from a progressive loss of dopaminergic neurons in the substantia nigra, motor symptoms mainly encompass bradykinesia, rigidity, and tremor (Damier, Hirsch, Agid, & Graybiel, 1999). Frequent additional nonmotor symptoms include depression, anxiety, apathy, (hypo‐)mania, dementia, psychosis, and impulse control disorder (Callesen, Weintraub, Damholdt, & Moller, 2014; van der Hoek et al., 2011; Maier et al., 2014; Reijnders, Ehrt, Weber, Aarsland, & Leentjens, 2008; Richard, 2007; Riedel et al., 2008; Sagna, Gallo, & Pontone, 2014; Starkstein, Brockman, & Hayhow, 2012). Together with motor symptoms (Appleman, Stavitsky, & Cronin‐Golomb, 2011; Hechtner et al., 2014; Weintraub et al., 2010), nonmotor symptoms can severely affect patients′ health‐related quality of life (Montel, Bonnet, & Bungener, 2009; Shearer, Green, Counsell, & Zajicek, 2012; for reviews see Den Oudsten, Van Heck, & De Vries, 2007; van Uem et al., 2016). Health‐related quality of life encompasses those aspects of well‐being and satisfaction with life that affect the individual's physical and mental health, including functional status, and social support as well as the subjective health perception (Centers for Disease Control and Prevention, 2000). Well‐being can be conceptualized as a frequent positive affect, an infrequent negative affect, and positive cognitive evaluations (Diener, 1984).

While disease‐related physical inactivity is discussed as an important factor for decreased quality of life (Cavanaugh et al., 2015; Wallen, Franzen, Nero, & Hagstromer, 2015), exercise has been suggested to improve patients′ well‐being (Ellis et al., 2011; for review and meta‐analysis see Goodwin, Richards, Taylor, Taylor, & Campbell, 2008). In this context, specific activating therapies, such as interdisciplinary rehabilitation (Monticone, Ambrosini, Laurini, Rocca, & Foti, 2015), self‐management programs (Tickle‐Degnen, Ellis, Saint‐Hilaire, Thomas, & Wagenaar, 2010), health promotion programs (Montgomery et al., 1994), physical activity training (for reviews see Foster, Bedekar, & Tickle‐Degnen, 2014), and dance (for a review see Sharp & Hewitt, 2014) have shown positive effects on both motor and nonmotor symptoms as well as on quality of life.

Clinically, it is therefore of interest whether these positive effects rely on specialized therapy programs, or if also the level of physical activity in everyday life mediates patients′ subjective condition. We hypothesized a mutual relationship between physical activity and the subjective condition as well as a modifying function of personal factors, such as coping strategies, personality traits, and disease symptoms.

We, therefore, asked PD patients to rate their subjective condition every two hours together with a self‐performed documentation of the physical activities they had performed during each preceding two‐hour interval. To detect bidirectional relations between the subjective condition and activities, we performed stepwise multivariate linear regression analyses (MRAs) (i) between the subjective condition and the duration of subsequent physical activities as well as (ii) between physical activities and the subsequent change in condition. The resulting significant relationships were entered into a subsequent MRA in order to assess possible associations with personal factors.

## 2 MATERIAL AND METHODS

### 2.1 Participants

Participants were recruited from the neurology outpatient clinic for movement disorders of the Charité University Hospital in Berlin (Germany) based on predefined inclusion and exclusion criteria. Inclusion criteria were a diagnosis of PD according to the Brain Bank Criteria as assessed by a specialist for neurology, optimized and stable antiparkinsonian medication, and current PD motor symptoms expressed by a rating above 10 points on the Movement Disorder Society‐sponsored revision of the Unified Parkinson's Disease Rating Scale (*MDS‐UPDRS*) (Goetz et al., 2008) part III.

Exclusion criteria were a diagnosis of other neurological disorders that were unrelated to the PD diagnosis as well as dementia, psychosis, mania, or severe motor impairment including use of a wheelchair, or *MDS‐UPDRS part III* >35. The recruitment interval was November 2015 to March 2016.

All participants gave written informed consent to the study protocol approved by the local ethics committee (protocol number EA4/134/15). The study has been performed in accordance with the ethical standards as laid down in the Declaration of Helsinki.

### 2.2 Method

Clinical assessments were performed by the treating neurologist, specialized in neurological movement disorders, using the *MDS‐UPDRS* examiner rating to evaluate typical PD symptoms (score from 0 to 180 points, duration about 20 min) and the NonMotor Symptom assessment scale for Parkinson's Disease (*NMS*) (Chaudhuri, Yates, & Martinez‐Martin, 2005) to quantify PD‐related nonmotor symptoms (score from 0 to 480 points, duration about 5 min). The Hamilton Rating Scale for Depression (*Ham‐D*) (Hamilton, 1960) was administered by a specialist for psychiatry to evaluate comorbid depressive symptoms (score from 0 to 50 points, 8–13 =  mild depression; 14–18 =  moderate depression; 19–22 =  severe depression; ≥23 =  very severe depression, duration about 15 min) and the Parkinson neuropsychometric dementia assessment (*PANDA*) (Kalbe et al., 2008) to assess PD‐related cognitive deficits (score from 0 to 30 points, duration about 15 min). Additionally, patients assessed themselves based on the (i) Parkinson's Disease Questionnaire (*PDQ‐39*) (Peto, Jenkinson, Fitzpatrick, & Greenhall, 1995) to evaluate disease‐specific functioning and quality of life (score from 0 to 100%, duration about 15 min), the (ii) Fatigue Severity Scale (*FSS*) (Krupp, LaRocca, Muir‐Nash, & Steinberg, 1989) to assess severity of disease‐related fatigue (score from 9 to 63 points, duration about 5 min), (iii) the self‐assessment part of the *MDS‐UPDRS* to quantify the subjective presence of motor and nonmotor symptoms (score from 0 to 80 points, duration about 10 min), (iv) the *brief‐COPE* (Knoll, Rieckmann, & Schwarzer, 2005) to assess the individual use of different coping strategies (0 to 8 points per dimension, duration about 10 min), and (v) the NEO Five‐Factor Inventory (*NEO‐FFI*) (Borkenau & Ostendorf, 2008) to evaluate the five personality factors neuroticism, extroversion, agreeableness, openness to experience, and conscientiousness (0 to 4 points per dimension, duration about 15 min). Moreover, caregivers evaluated impairments in goal‐directed behavior in four domains by the apathy evaluation score (*AES*) (Marin, 1991) (here expressed as ratio of each maximum subscore, duration about 5 min).

All participants were asked to evaluate their subjective condition during 2 weekdays of normal routine, that is, neither being significantly more active than usually nor significantly less active, every two hours of their waking time (nine assessment points per day) within 1 week after the above assessment. The evaluations were carried out for relevant motor and nonmotor PD symptoms on a ten point visual analog scale (0 =  not present, 10 =  maximum parameter value) for the parameters (i) *hypokinesia*, (ii) *hyperkinesia,* (iii) *happiness*, (iv) *sadness*, (v) *anxiety*, (vi) *nervousness*, (vii) *motivation*, (viii) *concentration*, (ix) *sleepiness*, (x) *pain*. At each assessment time point, patients also indicated the duration of activities they had engaged in during the preceding two‐hour interval. The kind of activity was selected from a pick list based on the PRISCUS‐Physical Activity Questionnaire (PRISCUS‐PAQ) (Trampisch et al., 2010). The original PRISCUS‐PAQ is a standardized interview to assess the daily time elderly people spend at rest or perform different physical activities such as “household labor,” “sports,” and “garden work” that was developed using correlations with accelerometric data. In reference to earlier studies, physical activities can be clustered with respect to calorie consumption for activities above the individual resting metabolic rate to consume more energy than the resting state (Hall et al., 2014; Jette, Sidney, & Blumchen, 1990). We summarized activities as *physical rest*,* moderate physical activity*,* harder physical activity*, and *heavy physical work*, taking in consideration the estimated energy consumption of PD patients (for details see Appendix).

### 2.3 Statistics

To group subjective condition parameters, we performed a principle component analysis (PCA) including factors with an eigenvalue above one, using varimax rotation and the Kaiser‐Meyer‐Olkin (KMO) measure as well as Bartlett's test of sphericity to estimate data adequacy. This PCA (KMO: .637; Bartlett's test highly significant; 63% total variance explained) delivered three principle components (PCs): PC1 comprising *sleepiness*,* hypokinesia*,* nervousness*,* pain*,* anxiety*,* sadness* (in the following labeled as “*negative condition*”), PC2 comprising *happiness motivation,* and *concentration* (labeled as “*positive condition*”), and PC3 being analog to *hyperkinesia*.

Based on these data, the statistical analyses aimed to assess bidirectional relationships between the subjective condition and the duration of physical activities. To relate the condition to the following activities, we performed the first MRA using the *positive condition* as independent and the duration of subsequent *physical rest*,* moderate physical activity*,* harder physical activity*, and *heavy physical work* as dependent variables (expressed as the sum of the according activities within each two‐hour interval in minutes; for details see Appendix Table A1). Due to a strong negative correlation between the values of the *positive* and the *negative condition,* no separate analysis was performed using the *negative condition* as independent variable. Next, the relationship between activities and subsequent changes in the subjective condition was explored. For this purpose, the “*positive condition change score*” was defined as the individual *positive condition* after the corresponding two‐hour interval minus the individual *positive condition* before the interval_._ The “*negative condition change score*” was defined likewise. These change scores were then related to the duration of the respective activities by means of a second MRA including *physical rest*,* moderate physical activity*,* harder physical activity*, and *heavy physical work* as independent variables and *positive condition change score* as dependent variable. The same was performed regarding the *negative condition change score*.

We furthermore aimed to assess a possible influence of personal factors on the thus identified relationships between activities and the change in condition. We, therefore, clustered individual results of *NEO‐FFI*,* brief‐COPE*,* MDS‐UPDRS* part I, II, and III, *NMS*,* Ham‐D*, and *FSS* by means of PCA (labeled as “*personal factors‐PCA,*” KMO: .533; Bartlett's test significant; 60.0% total variance explained) which delivered four PCs (PC1 comprising *MDS‐UPDRS* part I and II, *FSS*,* Ham‐D*, an *PDQ‐39*; PC2 comprising “positive reframing,” “active coping,” “venting,” “planning,” “agreeableness,” “conscientiousness,” “openness to experience,” and “extroversion”; PC3 comprising *NMS* and the reciprocal value of “acceptance”; PC4 comprising “use of emotional support,” “use of instrumental support,” and the reciprocal value of *MDS‐UPDRS* part III).

Next, a third MRA was carried out using the above derived individual coefficients between activity duration and *condition change score* as dependent and the values of the *personal factors‐PCs* as independent variables.

Lastly, not to miss associations between further clinical scores and time spent actively, individual scores of MDS‐UPDRS (parts I, II, III, total), NMS, Ham‐D, PDQ‐39, FSS, PANDA, as well as age, disease duration, and LED were entered as independent variables into a fourth MRA with daily time spent physically active (i.e., sum score of activities) as the dependent variable.

## 3 RESULTS

### 3.1 General characteristics

Twenty‐one (seven female / fourteen male) PD patients participated in the study. All met inclusion and exclusion criteria. Overviews of the demographic and clinical data as well as of further clinical scores are provided in Tables 1 and 2. Patients were on average moderately affected by motor symptoms, two patients suffered from marked motor fluctuations (*MDS‐UPDRS part IV* 10 and 12 points). All patients received usual drug combinations of levodopa, dopamine agonists, MAO‐B, and/or COMT inhibitors. Five patients fulfilled the *Ham‐D* criteria of a mild and two of a moderate depression, of which one was treated with an antidepressant. Apathy was generally rated as low (*AES*: 0.79 ± 0.16 with 1 indicating no and 0 the maximum apathy symptoms (Marin, 1991).

**Table 1:** Demographic and clinical characteristics

	Mean ± *SD*
Age (years)	70.05 ± 9.45
Disease duration (years)	6.90 ± 5.56
MDS‐UPDRS total (points)	46.80 ± 18.89
Hoehn and Yahr (stage; median)	2.0
LED (mg)	390.02 ± 415.64
PANDA (points)	21.19 ± 5.60
Education (years)	10.93 ± 1.75

MDS‐UPDRS, Movement Disorder Society‐sponsored revision of the Unified Parkinson's Disease Rating Scale; LED, Levodopa equivalent dose; PANDA, Parkinson neuropsychometric dementia assessment.

**Table 2:** Further clinical scores

	Mean ± *SD*
MDS‐UPDRS I (nonmotor exp. of daily living) [0–52 points]	11.48 ± 6.05
MDS‐UPDRS II (motor aspects of exp. of daily living) [0–52 points]	9.71 ± 7.62
MDS‐UPDRS III (motor examination) [0–132 points]	22.95 ± 8.73
MDS‐UPDRS IV (motor complications) [0–24 points]	1.62 ± 3.43
NMS [0–480 points]	54.26 ± 28.03
Ham‐D [0–50 points]	8.00 ± 5.01
PDQ‐39 [0–100%]	21.33 ± 15.45
FSS [9–63 points]	28.81 ± 14.01

*MDS‐UPDRS*, Movement Disorder Society‐sponsored revision of the Unified Parkinson's Disease Rating Scale; *NMS*, NonMotor Symptom assessment scale for Parkinson's Disease; *Ham‐D*, Hamilton Rating Scale for Depression; *PDQ‐39*, Parkinson's Disease Questionnaire; *FSS*, Fatigue Severity Scale; Higher values indicate higher symptomatology.

The *positive condition* (i.e., happiness, motivation, and concentration) was rated on average as 5.31 (±0.69) points and the *negative condition* (i.e., sleepiness, hypokinesia, nervousness, pain, anxiety, and sadness) as 2.60 (±0.28) points on a ten point scale.

**Figure 1:**
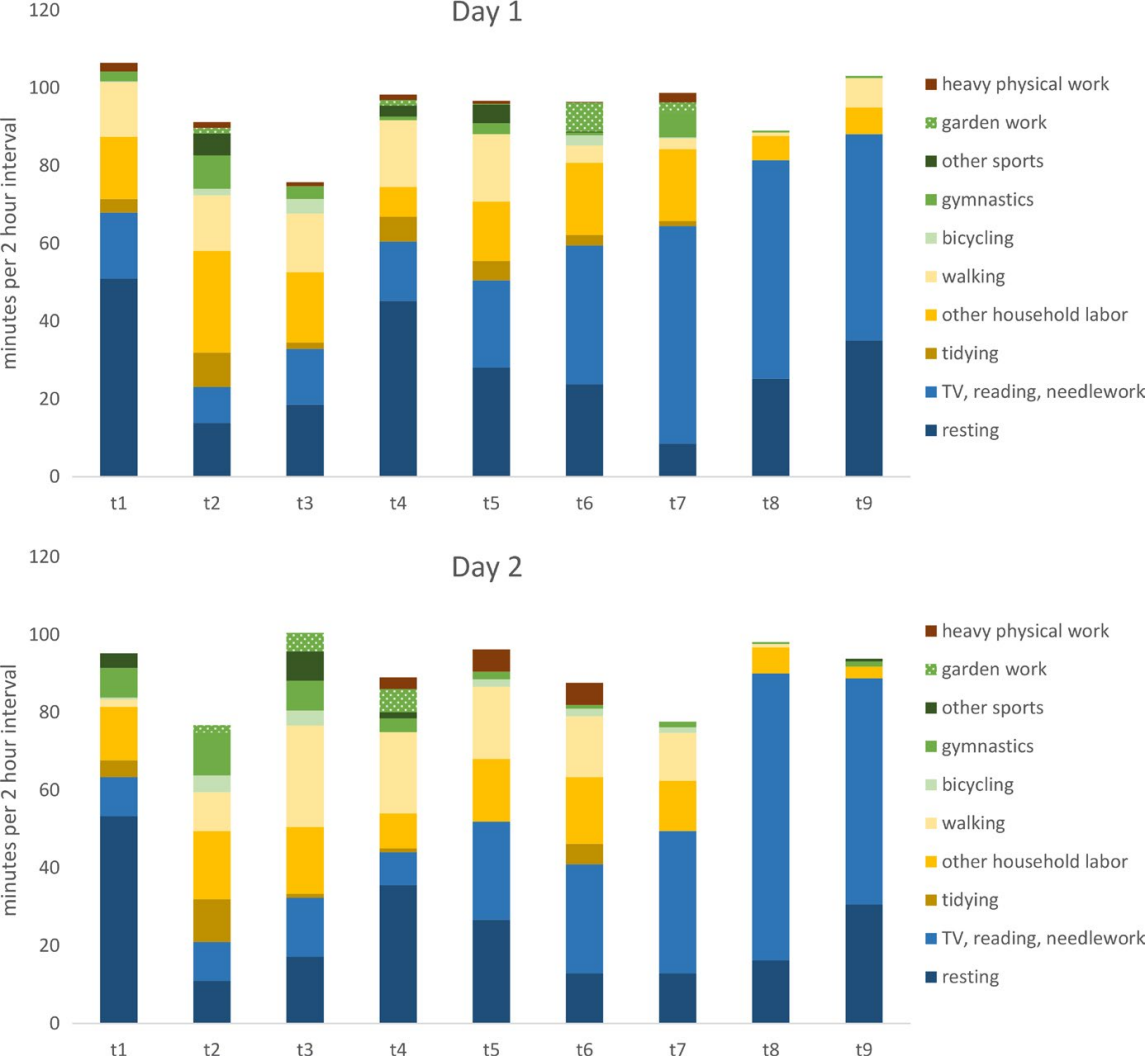
Distribution of Activities per Day. The average distribution of activities is depicted for nine 120‐min interval throughout 2 weekdays (day 1 and day 2). The abscissa reflects the respective assessment points (t1 to t9) following each 120‐minute interval. The ordinate indicates time in minutes. Blue areas represent physical rest (resting, watching TV, reading, or doing needlework), yellow areas moderate physical activities (tidying, other household labor, walking outside), green areas harder physical activities (bicycling, doing gymnastics, practicing other sports, doing garden work), and brown areas heavy physical work (chopping wood, shoveling snow). Due to the option of marking “other” (i.e., activities not specified by the pic list) the columns do not add up to 120 min

Figure 1 provides a detailed overview of the distribution of the activities per day. Participants reported to spend on average 8.59 (±2.93) hours per day at physical rest, 4.12 (±2.47) hours moderately active (e.g., doing household labor or walking outside), 1.13 (±1.08) hours performing harder activities (e.g., practicing sports or doing garden work), and 0.22 (±0.67) hours performing heavy physical work (e.g., chopping wood or shoveling snow).

### 3.2 Regression analyses

The first MRA indicated the *positive condition* as a highly significant predictor for the duration of subsequent *moderate physical activity* and as a significant predictor for the duration of subsequent *harder physical activity* (see Table 3, MRA 1).

**Table 3:** Multivariate regression analyses

MRA	Variables entered		Model summary			
	Independent	Dependent	*p*	β	adj *r* ^2^	*SE*
1	Positive condition	Moderate activity	<.001	0.841	.689	0.237
		Harder activity	.011	0.585	.301	0.164
2	Moderate activity	Positive condition change score	.001	0.759	.545	0.088
3	Personal factors‐PC2	Coefficient MRA 2	.009	0.651	.380	0.048
4	NMS	Time spent active	.013	−0.560	.273	0.022

*adj r* ^*2*^: adjusted *r* ^2^; ß: standardized coefficient beta; *MRA*: multivariate regression analysis; *NMS*: NonMotor Symptom assessment scale for Parkinson's Disease; *PC*: principal component, *SE*: standard error. *Personal factors‐PC2* comprised “positive reframing,” “active coping,” “venting,” “planning,” “agreeableness,” “conscientiousness,” “openness to experience,” and “extroversion.”

The table shows model summaries for significant relationships between entered independent and dependent variables of four stepwise multivariate linear regression analyses (MRAs). MRA 1 investigated the relationship between the positive condition (independent variable) and the duration of subsequent activities (dependent variables). MRA 2 investigated the relationship between the duration of activities (independent variables) and the positive condition change score (dependent variable). MRA 3 investigated the relationship between personal factors expressed as principal components (PC, independent variables) and the individual coefficient from MRA 2 (dependent variable), MRA 4 investigated the relationship between clinical scores (independent variables) and the daily time spent physically active (dependent variable).

The second MRA indicated the duration of *moderate physical activity* as a highly significant predictor for a subsequent increase in positive condition, expressed by the *positive condition change score* (see Table 3, MRA 2). With respect to the *negative condition change score*, none of the independent variables reached the level of significance.

The third MRA delivered a significant positive relationship between the individual coefficients of the association between *moderate physical activity* and *positive condition* and PC2 of the *personal factors‐PCA* which included the coping factors “positive reframing,” “active coping,” “venting,” “planning” as well as the personality factors “agreeableness,” “conscientiousness,” “openness to experience,” and “extroversion” (see Table 3, MRA 3).

Finally, the fourth MRA showed a weak negative relationship between the daily time spent physically active and the *NMS* (see Table 3, MRA 4). None of the other independent variables reached the level of significance.

## 4 DISCUSSION

In the current work, we studied interactions between the subjective condition and daily activities of PD patients as well as potential influences of personality and disease‐related factors. The results suggest an association between a positive subjective condition (i.e., happiness, motivation, and concentration) and the duration of subsequent physical activities. At the same time, the duration of moderate physical activity was significantly associated with an improvement in the subjective condition. This effect was pronounced in patients who mainly used *positive reframing* and *active coping* strategies and predominantly showed personality traits such as *agreeableness, conscientiousness, openness,* and *extroversion*. Worthwhile noticing, the daily time spent physically active was related to nonmotor rather than motor symptom severity or disease duration.

Our findings can thus be interpreted as a positive feedback process between physical activity and the subjective condition across different stages of disease progression.

Regarding temporal aspects, a minimum of about half an hour of moderate physical activity was associated with an improvement in the subjective condition. In view of the intensity, only moderate (such as household labor or walking outside) but not harder physical activity (such as bicycling, gymnastics, or garden work) was significantly related to a subsequent improvement.

In view of psychological and personality factors, previous studies have related higher quality of life to planful problem solving (Bucks et al., 2011; Montel et al., 2009), whereas escape‐avoidance behavior and high values of neuroticism have been associated with poor mobility and well‐being in PD patients (Whitworth et al., 2013; cf. Hurt et al., 2012). Furthermore, although activity decreases with disease duration (Cavanaugh et al., 2015), self‐efficacy rather than disability has previously been proposed as a main predictor for patients’ participation in regular exercise (Ellis et al., 2011). Negative expectations, on the other hand, are believed to constitute significant impediments to activity (Ellis et al., 2013). The current data add to these findings in that active coping strategies and personality traits appear to increase positive effects of physical activities on subjective well‐being.

In addition to the beneficial long‐term benefits of physical exercise (Ellis et al., 2011; Monticone et al., 2015; Sharp & Hewitt, 2014; Tickle‐Degnen et al., 2010; for reviews see Foster et al., 2014; Goodwin et al., 2008; Schenkman et al., 2012), the results point to almost immediate interactions between periods of activity in daily life and the subjective condition. Patients should therefore be encouraged to increase their activity levels by means of specialized programs as well as on an everyday basis. In this regard, external cueing appears important, particularly in depressive patients who are at risk of reinforcing their negative emotional state via inactivity (van der Hoek et al., 2011; Reijnders et al., 2008; Riedel et al., 2008; Shearer et al., 2012). Particularly these patients might profit from previously described self‐efficacy programs (Ellis et al., 2011; Starkstein et al., 2012; Tickle‐Degnen et al., 2010; Troeung, Egan, & Gasson, 2014; for reviews see Armento et al., 2012; Fernie, Kollmann, & Brown, 2015). In a reverse conclusion, a negative feedback process should be expected between physical rest and a negative subjective condition, suggesting that inactivity, for example, due to sedative medication, should be avoided whenever possible (Cabrera et al., 2010; Fialova et al., 2005; Happe, Berger, & Investigators, 2001; Onda et al., 2015; Tholfsen, Larsen, Schulz, Tysnes, & Gjerstad, 2015). The fact that our analyses showed no significant results regarding changes in negative condition scores was most likely due to a floor effect, considering the generally lower values of respective ratings.

There are several limitations of the study. The data mainly rely on self‐evaluations and are thus subjective. Data from video monitoring or wearable accelerometers might have expanded the informational value. Further, interviews rather than self‐reports could have increased the comparability. Regarding sample size and study duration, the current observations should be treated like a pilot study. Future studies should therefore include a larger cohort for a longer time period and consider the use of supportive technical tools. Lastly, this observational study leaves open the question whether externally motivated activity can exert comparable positive changes as self‐motivated activities. Further studies using an experimental design including a treatment and a control group could help to outline respective effects.

*In conclusion,* self‐reported moderate everyday life physical activity went along with an immediate improvement in the subjective condition of PD patients. This association was most pronounced in patients using active coping strategies. The global level of activity appeared to be associated with nonmotor symptom severity. This emphasizes the meaningfulness of careful nonmotor exploration and reduction of sedative influences in PD. The results suggest beneficial effects of increasing patients’ activity levels.

## CONFLICT OF INTEREST

None of the authors has any conflict of interest with respect to the present work. The corresponding author received honoraria for advisory activities from AbbVie, and holds a grant from the German Research Foundation (Kl 1276/5).

## APPENDIX 1

### 1.1

**Table A1:** List of activities

Item	Category	Activity
1	Physical rest	Resting (sitting or lying, e.g., for nap)
2		Watching TV, reading, or doing needlework
3	Physical activity, moderate	Tidying
4		Other household labor
5		Walking outside
6	Physical activity, harder	Bicycling
7		Doing gymnastics
8		Practicing other sports
9		Doing garden work
10	Heavy physical work	Heavy physical work (e.g., chopping wood, shoveling snow)

The table provides an overview of the activity items that patients could select as well the categorization used for further statistical evaluations. Patients were furthermore able to mark the category “other activities.” The selection of activities was developed in reference to the PRISCUS‐Physical Activity Questionnaire by Trampisch et al. (2010).
